# Raman spectroscopy detects chemical differences between potato tubers produced under normal and heat stress growing conditions

**DOI:** 10.3389/fpls.2023.1105603

**Published:** 2023-02-23

**Authors:** Sanjeev Gautam, Rohini Morey, Nina Rau, Douglas C. Scheuring, Dmitry Kurouski, M. Isabel Vales

**Affiliations:** ^1^ Department of Horticultural Sciences, Texas A&M University, College Station, TX, United States; ^2^ Department of Biochemistry and Biophysics, Texas A&M University, College Station, TX, United States

**Keywords:** abiotic stress, high temperature, global warming, quality, *Solanum tuberosum* spp. *tuberosum L*, Raman spectoscopy

## Abstract

Potato is the most consumed vegetable worldwide. Potato tubers contain water, starch, proteins, minerals, and vitamins. The amounts of these chemicals depend on the cultivar and growing location. When potatoes are exposed to high temperatures during the growing period, tuber yield and quality are detrimentally affected; however, there is limited knowledge about the influence of high temperatures on tuber chemical composition. With temperatures rising around the globe, the reaction of potato cultivars to high temperatures is increasingly important, and heat-induced changes, including changes in the chemical composition of tubers, should be considered. The Texas A&M University Potato Breeding Program has been selecting potato clones under high-temperature conditions for many years. Several released cultivars are considered heat-tolerant based on high marketable yields and low internal and external tuber defects. In this study, we used Raman spectroscopy (RS), an analytical tool, to determine whether heat stress causes changes in the chemical composition of tubers of ten potato cultivars. RS is a non-invasive method that requires less time and labor than conventional chemical analysis. We found drastic changes in the intensities of vibrational bands that originate from carbohydrates in the spectra acquired from tubers of heat-stressed plants compared to tubers produced by potato plants grown under normal conditions. These results demonstrate that RS could be used as a replacement or complement to conventional chemical analysis to inspect the effect of heat stress on tuber chemical composition.

## 1 Introduction

Potato (*Solanum tuberosum* ssp. *tuberosum L.*) is a staple crop for many people worldwide and is the most widely consumed vegetable, with a per capita consumption of 32.4 kg/year (FAOSTAT, 2022). Potato tubers are a major source of starch, sugars, proteins, vitamins (Vit. C, Vit. B1, Vit. B3, Vit. B5, Vit. B6), potassium, and dietary fiber (McGill et al., 2013; Beals, 2019; Haverkort et al., 2022). Potatoes and root vegetables are the world’s third largest provider of carbohydrates after rice and wheat for human consumption (FAO/WHO, 1998; CIP, 2020). Potato is a very adaptable crop as it can grow from mountainous regions at high altitudes, with poor soil conditions, to coastal areas. From the center of domestication around the Titikaka lake (in northern Bolivia and southern Peru), cultivated potatoes have expanded to more than 156 countries worldwide (FAO, 2019). Globally, potatoes were planted on 16.494 million hectares in 2020 and produced 359.071 million tonnes of tubers, with a yield of 21.8 Mg/ha (FAOSTAT, 2022). The top producer country is China. Potato in the USA ranks fifth and represents a very important industry (3.6 billion US$ in 2020) (USDA, 2018).

Despite being a resilient plant, extreme environmental conditions such as excessive heat threaten potato tuber yield and quality. Compared to the preindustrial period (1850-1900), the earth’s temperature at the end of the current century is predicted to increase by 1-1.8°C (under a very low greenhouse gas emission scenario) or 3.3-5.7°C (under very high greenhouse gas emission scenario) (IPCC, 2021). High-temperature stress during the potato growing period can become a major problem for potato growers and consumers. Heat stress can affect marketable yields, tuber quality, market value, and nutritional benefits of potatoes. In particular, potato tuber yield and quality (external appearance, internal defects, and processing quality) are detrimentally affected when night temperatures go above 18°C (Bushnell, 1925; Haverkort and Verhagen, 2008). Tito et al. (2018) reported a reduction of potato production of 87-97% when grown at 1.3 and 2.6°C higher than usual maximum temperatures. Heat stress will likely be a more frequent event in the future. Potato yield loss due to heat stress is estimated to be between 18% and 32% by the end of 2050 (Hijmans, 2003). Around 12.5% of the current potato production regions are projected to shift (‘climatic shift’) in 2070 compared to the 1970-2000 period. Thus, to maintain the current production level, there is a need to increase production by 44 million tonnes from the current acreage, or bring 2.1 million hectares under potato production, given new climatic conditions (Fumia et al., 2022). Specific gravity, tuber dry matter, starch, and reducing sugars are important quality attributes in potatoes and are especially critical considerations in the case of processing market classes (Islam et al., 2022). Specific gravity is an indirect measure of potato solids and affects oil absorption in fried/processed products (Gould and Plimpton, 1985). High specific gravity (1.080 or higher) is desired in the processing industry (Stark, 2020). An increase of 0.005 specific gravity can enhance chip yield by 0.78% and reduce chip oil content 1.33% (Lulai and Orr, 1979). Specific gravity, which reflects tuber dry matter and starch content in potatoes, is affected by environmental conditions and crop management. Lower specific gravity and, thus, lower dry matter and starch content are observed in potato tubers from plants grown under high-temperature conditions (Asmamaw et al., 2010). Reducing sugar levels below 0.35 mg/g of fresh weight (or <0.035%) is the benchmark for potatoes intended for chip production (Stark, 2020). Reducing sugars react with the amino acid asparagine to form acrylamide (a potential carcinogen) through the Maillard reaction. Thus, lowering the content of reducing sugars in potato tubers is desirable to minimize the amount of acrylamide produced in fried potato products. High temperatures during the potato growing season have also been associated with an increase in reducing sugars in tubers, which is highly undesirable (Eldredge et al., 1996).

It is challenging to bring new land into the production system and intensify production. An effective way of alleviating the detrimental effects of heat stress, whether short or long-term, is to develop heat-tolerant plant cultivars (Bonnel, 2008; Demirel et al., 2017; Busse et al., 2018; Gautam et al., 2021). Developing a tolerant cultivar begins with identifying promising germplasm that can provide quality yield so that its introgression into the potato gene pool is possible (Bashir et al., 2022). Texas is one of the US States where potato production is more severely affected by high-temperature stress resulting in challenges, especially for potato growers. However, the reality of growing potatoes under high-temperature stress offers the opportunity of selecting heat-tolerant potato varieties. The Texas A&M Potato Breeding Program has been selecting clones for the last 30-40 years under high-temperature conditions. Thus, it is plausible that Texas-bred potato clones should have some heat tolerance. The potato cultivars differ in their responses to high temperatures. The differential responses of potato cultivars to heat stress can be observed in changes in the chemical composition of the tubers under heat stress.

Currently, there are no technological or computational ways of analyzing the effect of heat stress on potatoes. Chemical tests are often used to measure the nutritional profile of a crop (Damodaran et al., 2017). Gravimetric analysis and Megazyme assays measure starch content (Zhu et al., 2008). Protein content in potatoes is usually quantified using the Dumas Combustion Method by analyzing the nitrogen content in the potato (Mihaljev et al., 2015). Although these tests are common in a laboratory setting, they are often destructive, time-consuming, labor-intensive, cannot be implemented in the field, and are not easily accessible to farmers. Near-infrared Spectroscopy has been used as a spectroscopic methodology for the chemical analysis of potatoes. However, such analysis cannot be done using fresh potatoes tubers due to their high water content (~80%) and thus requires freeze-drying the samples, which is destructive (Osborne et al., 1993; Berardo et al., 2004; Baranska et al., 2006; Kim et al., 2007; Stubbs et al., 2010).

Raman Spectroscopy (RS) is non-destructive and non-labor-intensive spectroscopic method based on inelastic light scattering (Kurouski et al., 2015). The inelastically scattered photons provide information about the sample’s chemical structure. Previous studies demonstrated that RS could diagnose potato disease (Farber et al., 2021), reveal nutritional profiles of potato tubers, and create chemical profiles of different potato cultivars (Morey et al., 2020). RS was also used to accurately identify soil nitrogen, potassium, or phosphorus deficiencies (Sanchez et al., 2020). In this study, we aim to investigate whether RS could be used to differentiate between potato clones grown under normal and heat-stressed conditions, as well as to determine which cultivars of potatoes were more affected by heat stress and which ones were heat-tolerant.

## 2 Materials and methods

### 2.1 Greenhouse experiments

#### 2.1.1 Plant materials

Ten potato cultivars (**Table 1**) were planted, grown, and harvested in greenhouses at the Horticulture Teaching Research and Extension Center at Texas A&M University, located near Somerville (Latitude: 30.5223 and Longitude: -96.4307), Texas, USA. Atlantic, Russet Burbank, Russet Norkotah and Yukon Gold are commercial cultivars used as references for different market classes. Atlantic is a chipping variety; Russet Burbank is a French fry processing potato; Russet Norkotah is a popular russet skin potato for the fresh market; Yukon Gold is a fresh market yellow cultivar. The Texas A&M University Potato Breeding Program released the other six potato clones used in the study. The Texas A&M potato breeding program has been selecting potato clones for the last 30-40 years under high-temperature conditions where several days with temperatures beyond 35°C are common (Vales et al., 2019). Thus, the clones from Texas A&M can be considered to possess heat tolerance characteristics. COTX09022-3RuRe/Y was released under the experimental code; it has russet skin, red eyes and yellow flesh. TX1523-1Ru/Y (Sierra Gold™) has russet skin and yellow flesh. Reveille Russet, Russet Norkotah 278, Russet Norkotah 296, and Vanguard Russet are fresh market potatoes with russet skin and white flesh.

**Table 1:** Tuber characteristics and plant maturity of the ten potato clones planted under two greenhouse conditions (normal vs. heat stress) in 2020 and 2021.

Clone	Codes	Tuber flesh color	Tuber skin type	Market class	Plant maturity
Atlantic	AT	White	Smooth (light russeting)	Processing (Chipping)	Medium
COTX09022-3RuRE/Y	CO	Yellow	Russet	Dual	Early-Medium
Reveille Russet	RR	White	Russet	Fresh	Late
Russet Burbank	RB	White	Russet	Processing (French fries)	Medium
Russet Norkotah	RN	White	Russet	Fresh	Medium
Russet Norkotah 278	RN278	White	Russet	Fresh	Medium
Russet Norkotah 296	RN296	White	Russet	Fresh	Medium
Sierra Gold™	SG	Yellow	Russet	Fresh	Early
Vanguard Russet	VR	White	Russet	Fresh	Medium-late
Yukon Gold	YG	Yellow	Smooth	Fresh	Early

#### 2.1.2 Experimental design

Potatoes were planted in a factorial block design in experiments conducted in 2020 and 2021. Potato tuber seed pieces (~56.7 g) of ten clones were planted in two greenhouses (one under normal growing conditions and the other under heat-stress conditions). Four replications were used for each clone and growing condition. Each replication had three plants, and each plant was in an 11.4 cm^3^ pot.

#### 2.1.3 Growth conditions

Sprouted seed pieces were planted in pots at a depth of ~10 cm from the surface. The pots were filled with ProMix BX (Premier Tech, Quakertown, PA) amended with the starter fertilizer Osmocote (Scotts Miracle-Gro, Marysville, OH) 50 g per pot. The greenhouses were set at 25/15°C day/night temperatures for the first 30 days. For the rest of the time, one greenhouse was maintained at 25/15°C day/night (normal conditions), whereas the other greenhouse was set to 35/25°C day/night (heat stress conditions). Extreme temperatures were recorded (**Supplementary Figure 1**). The greenhouse conditions were controlled with GROWCOM systems (Microgrow, Temecula, CA, USA). The greenhouses were cooled with evaporative cooling and heated with vented forced air propane heaters. External weather conditions affected greenhouse temperature control; it was difficult to cool the greenhouses when it was hot and humid outside. The greenhouses had warmer temperatures in 2021 than 2020 (**Supplementary Table 1**; **Supplementary Figure 1**). No artificial light was used during the experiment; recorded natural light conditions indicated that plants experienced long day conditions (14/10 hrs. day/night).

The tuber seed pieces were planted on February 24, in both years- 2020 and 2021. The plants were grown for about 90 days when the vines were killed. Tubers were left for ten days in the pots to ensure good skin set before harvesting. The tubers were harvested on May 27, 2020, and June 3, 2021. The specific gravity of tubers was evaluated by comparing the weight of tubers (~1kg, US No. 1 grade) in the air to the weight of the same volume of water using the following formula: specific gravity = [weight in air/(weight in air – weight in water)]. The four largest tubers from each replication were sampled for further analysis (Raman spectroscopy and wet chemistry). Tubers were stored at room temperature (20°C and 70% RH) in the dark until they were scanned with a Raman Spectrometer.

Raman analysis was performed for the 2020 and 2021 experiments on the same tubers used for wet chemistry. After the scans were completed, four tubers per replication were cut longitudinally from stem to bud end to generate four quarters per tuber; four quarters (a single quarter per tuber) were chopped and mixed thoroughly. About 15 g of chopped fresh tuber samples were weighed in 50 mL Falcon tubes. To calculate dry matter (DM), each sample’s fresh weight (FW) was obtained and immediately frozen at -20°C and later transferred to – 80°C for a few days before freeze-drying. The samples were freeze-dried (LABCONCO, FreeZone console freeze dryer 6L −50°C Series, Kansas City, MO, USA) with a collector temperature of (-50°C) and vacuum pressure of 0.21mbar for five days. Freeze-dried samples were weighed to obtain dry weight (DW), and DM was calculated using the formula: DM% = (DW/FW) *100. The freeze-dried potato samples were ground and homogenized with a grinder (1600 Mini G from SPEX^®^ SamplePrep, NJ, USA) at 1500 rpm for 1.5 minutes. Ground samples (about one gram per replication) were used to quantify protein. The protein content (in percentage of DW) was calculated by multiplying the total nitrogen obtained by the Kjeldahl method (McGeehan and Naylor, 1988) by a factor (6.25). Triplicates of five grams of freshly chopped tuber samples were taken for estimating reducing sugars. Reducing sugars were evaluated following the modified 3,5-Dinitrosalicylic acid (DNS) method (Gonçalves et al., 2010).

### 2.2 Raman spectroscopy

A hand-held Agilent Resolve spectrometer (Agilent Technologies, USA) equipped with 830 nm laser was used to obtain spectra from the potatoes. Raman spectra were collected from four tubers per clone (10 clones), per replication (4), and condition (2) (normal *vs*. heat stress) (2020, 2021). Each tuber was scanned a minimum of three times, resulting in 48 spectra per clone per greenhouse condition (normal *vs.* heat stress), which resulted in over nine hundred and sixty acquired spectra. The instrument collects three types of spectra – ‘surface’, ‘offset’ and ‘spatially-offset Raman spectra (SORS)’ (Matousek et al., 2005). In 2020, ‘offset’ spectra were obtained directly from the intact tubers (with skin on). We acquired spectra with good signal-to-noise ratios from tubers with thin skin. However, spectra with lower signal-to-noise ratios were obtained from tubers with heavy russet skin. To improve the signal-to-noise ratio in the acquired spectra, in 2021, a very small section (1cm^2^) of the skin of potato tubers was peeled (2 mm thickness) before taking ‘surface’ spectra. The spectral acquisition time for each scan was 1 s.

### 2.3 Statistical analysis

Analyses of variance for specific gravity, dry matter, reducing sugars, and protein traits were analyzed using the mixed model approach in JMP ^®^16 (SAS Institute Inc, 2021). Clones and growing conditions were considered fixed effects, whereas replications were random. Mean comparisons were made based on Tukey’s HSD. The results (**Supplementary Table 2**) were graphically represented using MS excel. Variances were calculated for each source of variation from ANOVA table using the sum of squares.

MATLAB equipped with PLS Toolbox (Eigenvector Inc., WA, USA) was used to perform partial least-squares discriminant analysis (PLS-DA) for all collected spectra. Four preprocessing steps were performed before analyzing the spectra for differences in the Normal and heat-stressed samples: (a) MSC Mean, (b) Smoothing (1^st^ polynomial order and 15 filter width), (c) 2^nd^ Derivative of the Spectra (3^rd^ Polynomial order and 51 filter width), (d) Normalization to the Area. Model was built with 70% of the acquired spectra which was then used to test 30% of the spectra to determine the true positive rate (TPR) for each model (normal growth vs. heat stress).

Analyses of variance (ANOVA) for the acquired Raman spectra were performed using MATLAB (MATLAB Co., USA). We focused on vibrational bands with the following peaks: a peak at 479 cm^-1^ to assess starch (Morey et al., 2020), a peak at 1527 cm^-1^ to inspect carotenoids (Adar, 2017), a peak at 1208 cm^-1^ representing phenylpropanoids (Larsen and Barsberg, 2010), and a peak at 1660 cm^-1^ for protein (Kurouski et al., 2015).

## 3 Results

### 3.1 Specific gravity

**Figure 1:**
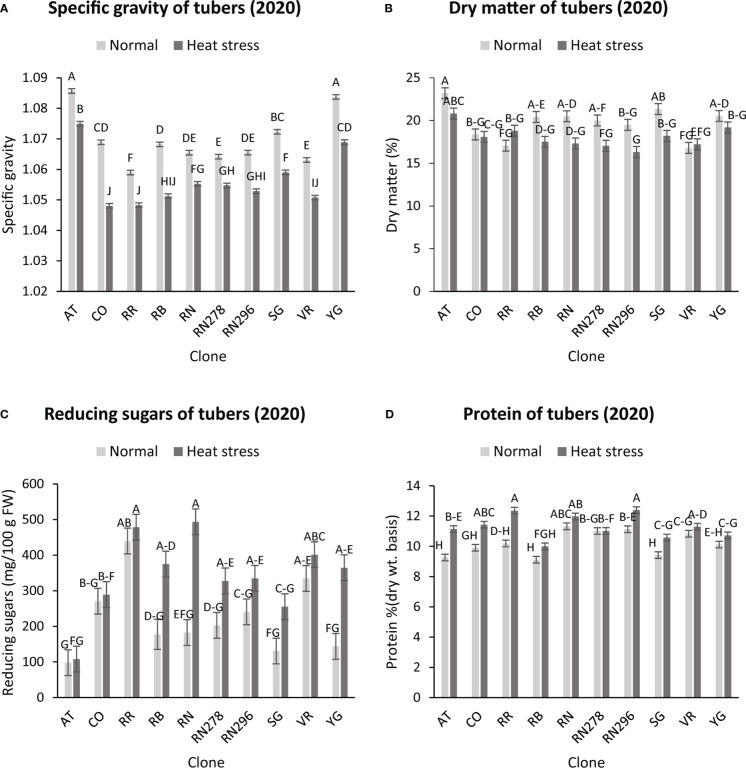
Effect of temperature and clone on different traits (2020). **(A)** Tuber Specific gravity, **(B)** Tuber dry matter **(C)** Total reducing sugars (glucose + fructose) in tubers, **(D)** Protein (% Dry weight basis). The following potato clones were used in the experiment: AT (Atlantic), CO (COTX09022-3RuRE/Y), RR (Reveille Russet), RB (Russet Burbank), RN (Russet Norkotah), RN278 (Russet Norkotah 278), RN296 (Russet Norkotah 296), Sierra Gold (SG), Vanguard Russet (VR) and Yukon Gold (YG). Bars with the same letter were not significantly different at p≤ 0.05.

**Figure 2:**
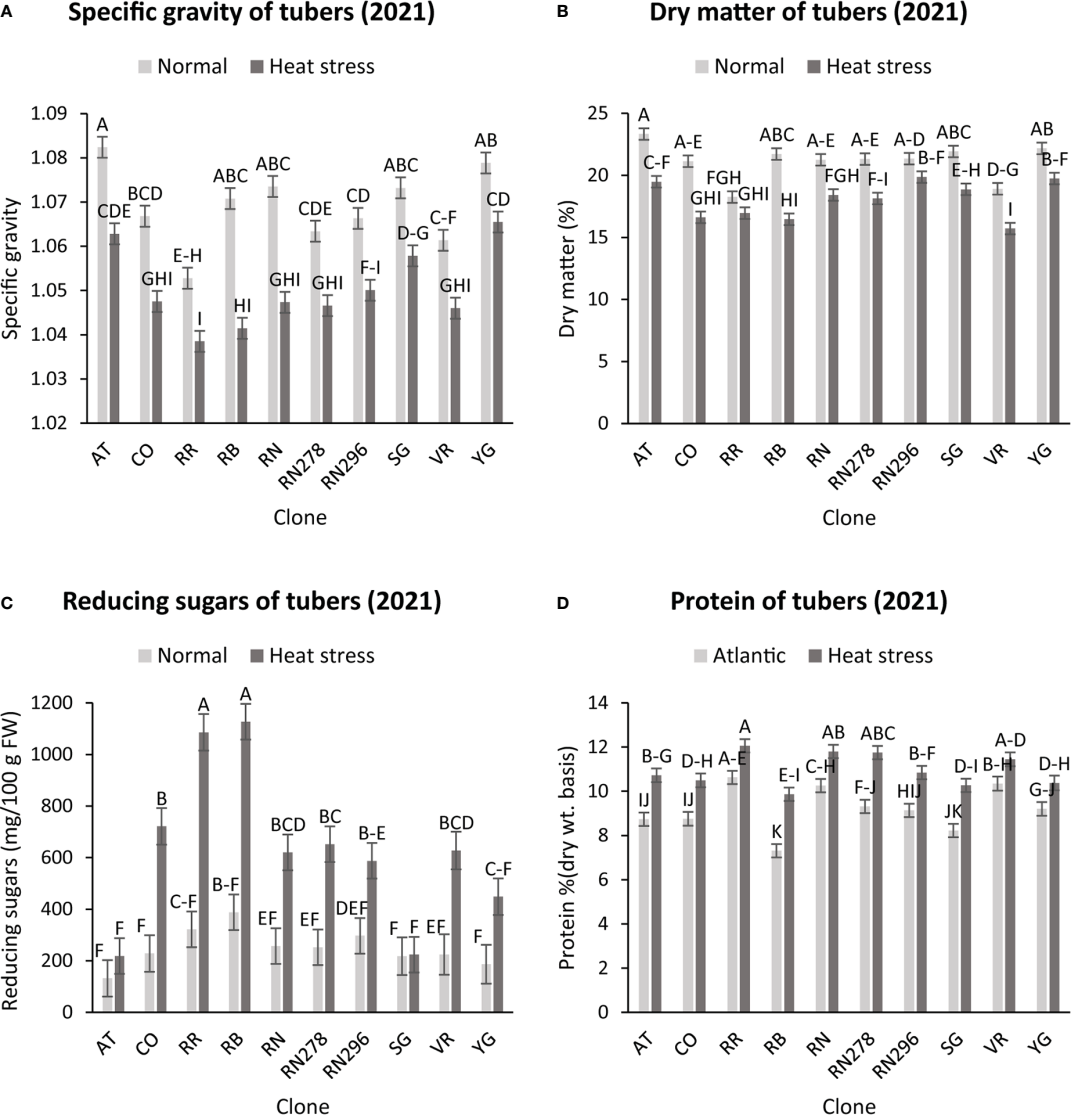
Effect of temperature and clone on different traits (2021). **(A)** Tuber Specific gravity, **(B)** Tuber dry matter **(C)** Total reducing sugars (glucose + fructose) in tubers, **(D)** Protein (% DW). The following potato clones were used in the experiment: AT (Atlantic), CO (COTX09022-3RuRE/Y), RR (Reveille Russet), RB (Russet Burbank), RN (Russet Norkotah), RN278 (Russet Norkotah 278), RN296 (Russet Norkotah 296), Sierra Gold (SG), Vanguard Russet (VR) and Yukon Gold (YG). Bars with the same letter were not significantly different at p≤ 0.05.

The highest specific gravity was observed in Atlantic under normal conditions in both years; this was expected since Atlantic is a chipping variety and high specific gravity (high starch content) is a required trait for this market group. Tubers produced under heat stress conditions had significantly lower specific gravities than those harvested from normal growing conditions (**Figures 1**, **2**). The interaction of clone*condition was significant, indicating a differential response of some clones to the growing conditions (**Figures 1**, **2**; **Table 2**). Potato tubers harvested from heat-stress greenhouse growing conditions had, on average, 1.2% and 1.7% lower specific gravity than those tubers produced under normal conditions in 2020 and 2021, respectively (**Table 3**). The least reduction (0.9%) was observed in Russet Norkotah 278, whereas the largest reduction (1.9%) was observed in COTX09022-3RuRE/Y followed by Russet Burbank (1.6%) in 2020. In 2021, tubers of Yukon Gold were the least affected by specific gravity reduction (1.2%).

**Table 2:** Analysis of variance for effect of different temperature conditions (normal vs. heat stress) on ten potato clones.

Source of Variation	Specific gravity	Tuber dry matter (%)	Reducing sugars (mg/100g)	Protein (% DW)
A. Year 2020				
Clone	***	***	***	***
Condition	***	ns	ns	**
Clone*Condition	***	**	***	***
Rep [Condition]	***	**	***	***
B. Year 2021				
Clone	***	***	***	***
Condition	***	**	**	***
Clone*Condition	***	***	***	*
Rep [Condition]	*	**	***	***

ns, non-significant; * p< 0.05, ** p< 0.01, *** p< 0.001. Four replications per clone per condition were used.

**Table 3:** Percent change in selected traits of clones grown under heat stress vs. normal growing conditions in greenhouses for year 2020 and 2021.

	Specific gravity		Tuber dry matter		Reducing sugars		Protein	
Clone	2020	2021	2020	2021	2020	2021	2020	2021
**Atlantic**	-1.0	-1.8	-10.2	-16.5	10.1	65.7	20.3	23.8
**COTX09022-3RuRE/Y**	-1.9	-1.8	-1.6	-21.4	6.9	216.3	15.4	20.0
**Reveille Russet**	-1.0	-1.4	10.2	-7.1	8.8	237.5	21.3	13.1
**Russet Burbank**	-1.6	-2.7	-14.3	-24.2	111.1	190.3	9.8	34.9
**Russet Norkotah**	-1.0	-2.4	-15.6	-13.3	170.3	141.2	5.7	15.1
**Russet Norkotah278**	-0.9	-1.6	-14.8	-14.9	61.6	158.4	0.1	26.2
**Russet Norkotah296**	-1.2	-1.5	-16.3	-7.0	39.1	98.2	11.3	18.7
**Sierra Gold**	-1.2	-1.4	-14.7	-13.9	95.2	2.7	12.3	24.9
**Vanguard Russet**	-1.2	-1.4	2.5	-16.9	20.0	179.9	4.3	11.5
**Yukon Gold**	-1.4	-1.2	-6.6	-11.0	153.8	140.4	6.0	13.3
**Average**	-1.2	-1.7	-8.1	-14.6	67.7	143.0	10.6	20.2

^*^Negative values indicate percent reduction in the trait from normal to heat stress conditions, whereas positive values indicate percent increase in the trait from normal to heat stress conditions. Calculated with the formula: % change= (Value at Heat stress- Value at Normal condition)/Value at Normal condition*100.

**Figure 3:**
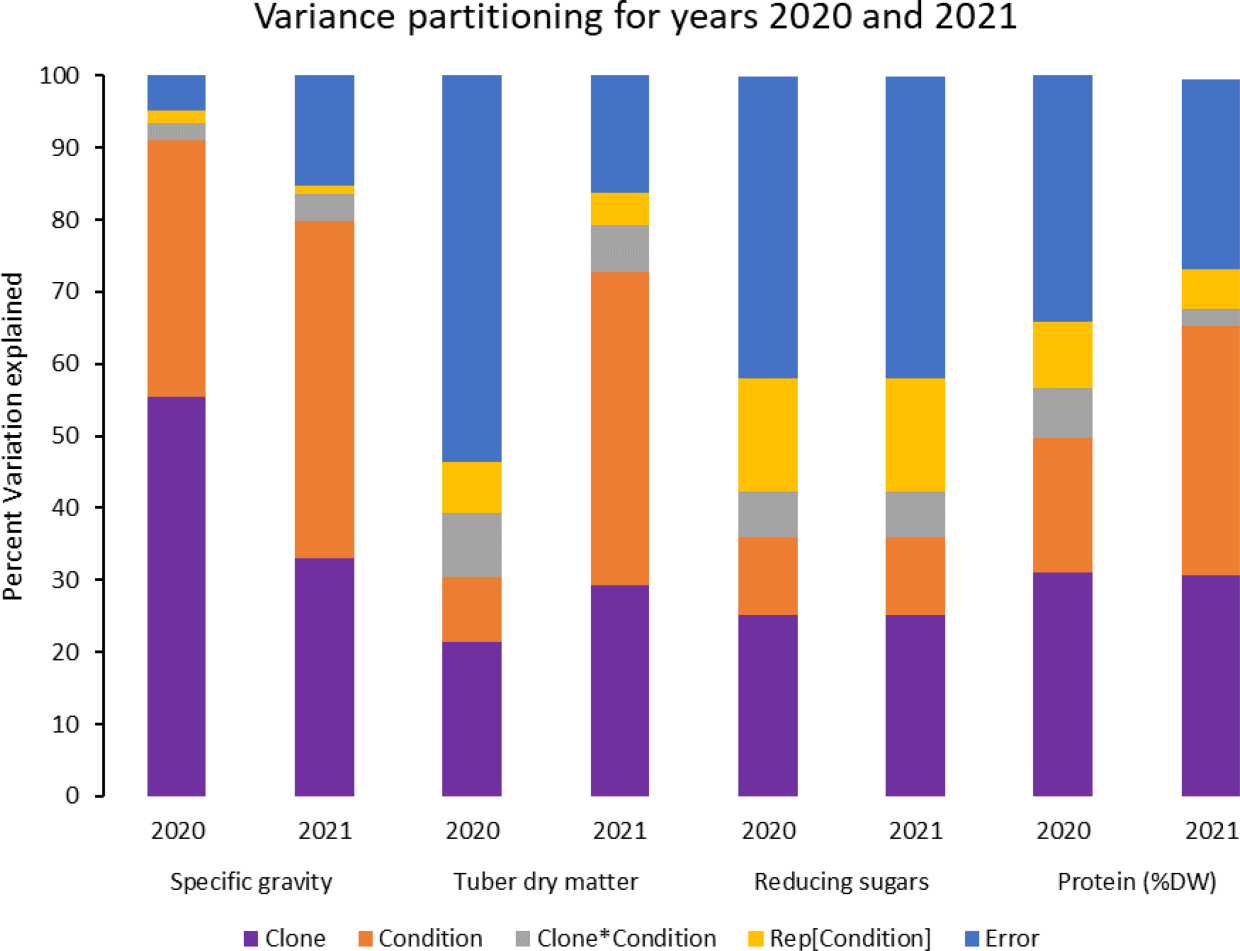
Phenotypic variance explained for some selected traits in potatoes grown under normal vs. heat stress conditions (2020 and 2021).

In contrast, tubers of Russet Burbank were the most affected by specific gravity reduction (2.7%) due to high-temperature stress. Conditions and clones alone explained more than one-third variation associated with specific gravity in both years (**Figure 3**). The interaction between clone and condition explained from 2.3% to 4% variation in the specific gravity of potato tubers harvested in 2020 and 2021.

### 3.2 Tuber dry matter

Similar to specific gravity, tubers produced under heat stress conditions had significantly lower tuber dry matter than those harvested from normal growing conditions. Clones showed differential responses for tuber dry matter under different temperature conditions (**Figures 1**, **2**; **Table 2**). Tuber dry matter is highly correlated with specific gravity, and Atlantic had the highest tuber dry matter. Variation in tuber dry matter was associated with clones, conditions, and interaction between clones and conditions in decreasing order in both years (**Figure 3**). Though a significant interaction was observed for tuber dry matter, the genetic makeup of the clones had a strong influence, as well as the growing conditions of the plants. The potato clones had an 8.1% (2020) and 14.6% (2021) reduction of tuber dry matter when grown under high-temperature conditions as compared to the normal growing condition (**Table 3**). The largest reduction in tuber dry matter (16.3%) was observed in Russet Norkotah 296 followed by Russet Norkotah (15.6%) and Russet Burbank (14.3%) whereas tuber dry matter increased by 2.5% and 10.2% in Vanguard Russet and Reveille Russet, respectively in 2020. In 2021, the highest reduction of tuber dry matter was observed in Russet Burbank (24.2%), while the lowest reduction was observed in Russet Norkotah 296 (7.0%) together with Reveille Russet (7.0%).

### 3.3 Reducing sugars

Reduced sugars (glucose + fructose) in tubers were significantly higher under heat-stress growing conditions than under normal growing conditions. There was an increase of 67.7% (2020) and 143.0% (2021) in reducing sugars in tubers grown under heat stress compared with that of tubers grown under normal temperature conditions (**Table 3**). Under normal conditions, the lowest reducing sugars were observed in Atlantic followed by Sierra Gold and Yukon Gold in 2020 and Atlantic, Sierra Gold, Yukon Gold and COTX09022-3RuRE/Y in 2021. As in the case of specific gravity and tuber dry matter, reducing sugars also exhibited significant clone-by-condition interaction (**Figures 1**, **2**), however, the interaction effect (clone*condition) for reducing sugars was lower (**Figure 3**). Variation in reducing sugars could thus be attributed mainly to the genetic makeup of the clone and the growing condition in which the potatoes were grown (**Figure 3**).

### 3.4 Protein

Protein (% DW) in tubers was significantly increased under heat stress conditions (**Figures 1**, **2**). Though there was a significant interaction between clones and growing conditions regarding protein in tubers, the effect size of the interaction (7% in 2020 and 2% in 2021) was too small to explain the variation. However, clone (31% in 2020 and 30% in 2021) and condition (19% in 2020 and 35% in 2021) explained most of the variation observed in protein percentage in the tubers from the experiment (**Figure 3**). Under heat stress conditions, Reveille Russet had the highest protein content in both years. The lowest protein content was observed in Russet Burbank and Sierra Gold under normal conditions. The increase in protein (dry weight basis) was found to be 10.6% in 2020 and 20.2% in 2021 (**Table 3**).

### 3.5 Raman spectra

**Figure 4:**
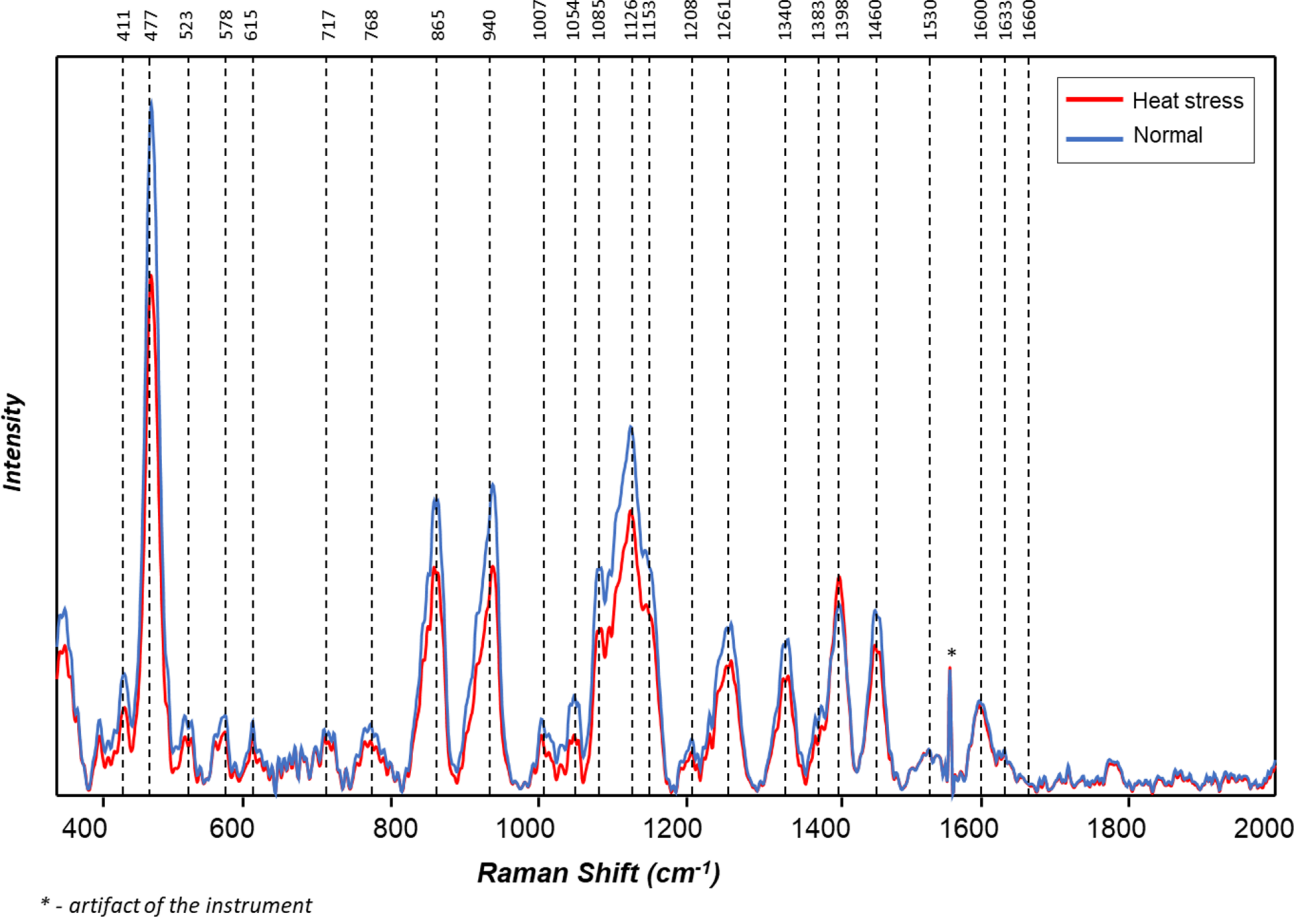
Averaged Raman spectra of tubers of ten potato cultivars grown under normal *vs.* heat stress greenhouse conditions (2020).

**Figure 5:**
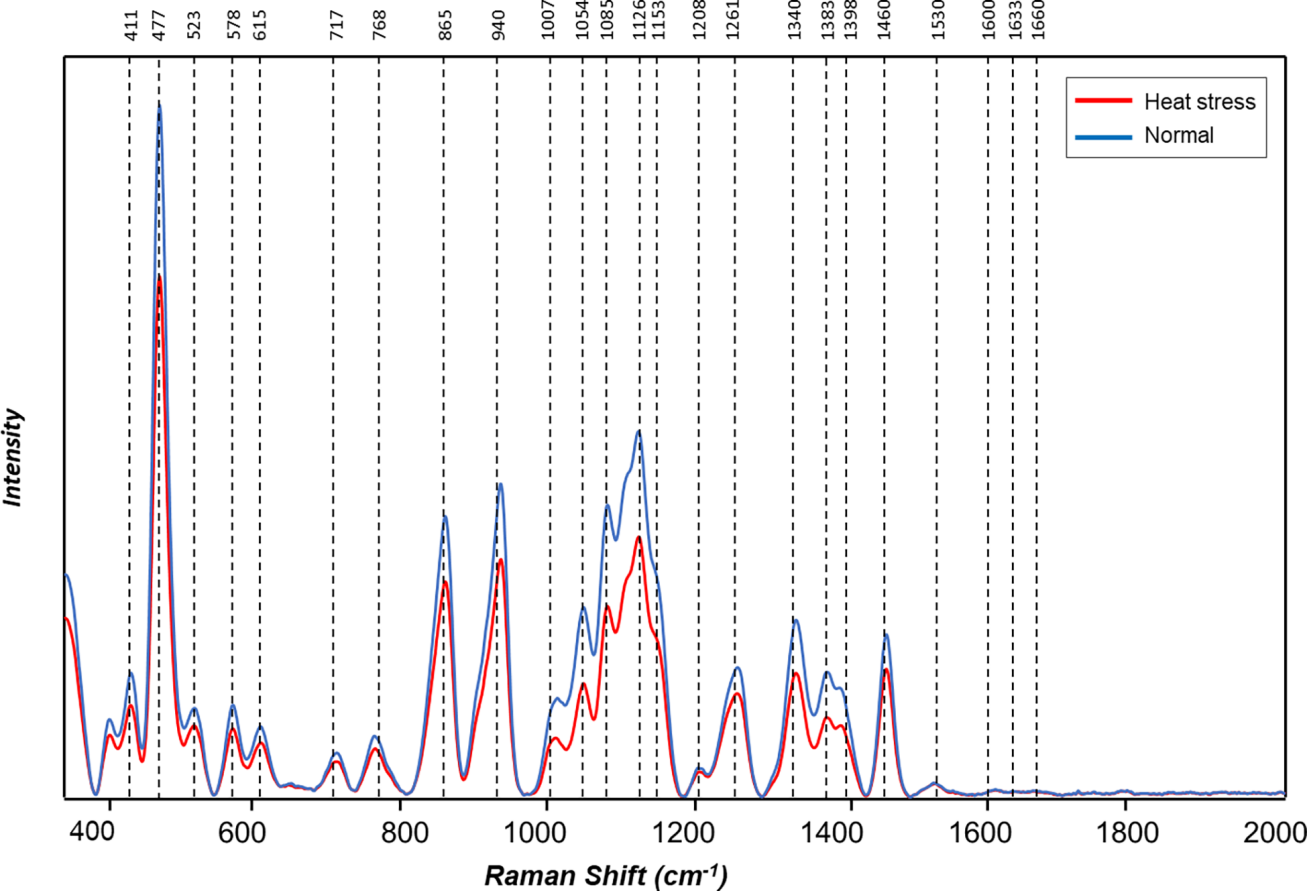
Averaged Raman spectra of tubers of ten potato cultivars grown under normal *vs.* heat stress greenhouse conditions (2021).

**Figure 6:**
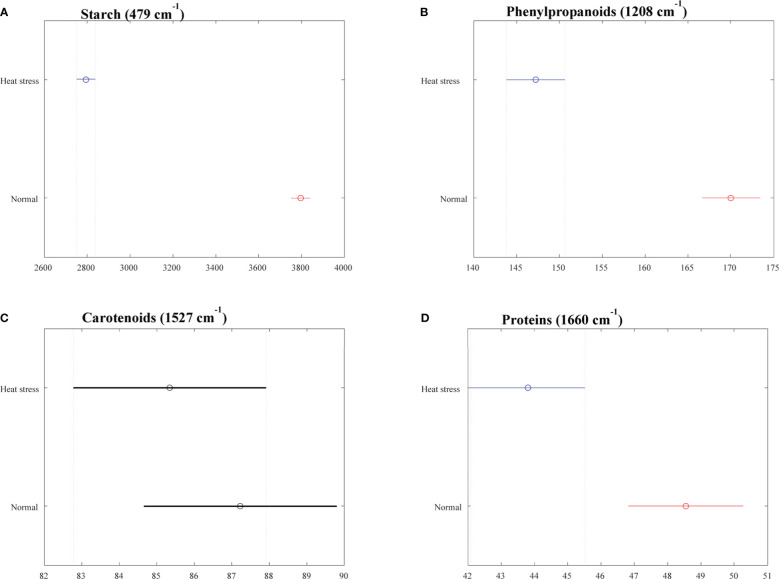
Selected vibrational bands correspond to different chemicals **(A)** Starch, **(B)** Phenylptopananoids, **(C)** Carotenoids, **(D)** Proteins in the tubers (2021). Means are indicated as a circle, and the bars indicate confidence intervals for the intensity (x axis) of the spectra. Statistically significant differences for a particular Raman band when comparing clones growing under normal *vs.* heat stress conditions were denoted with red and blue colors.

**Figure 7:**
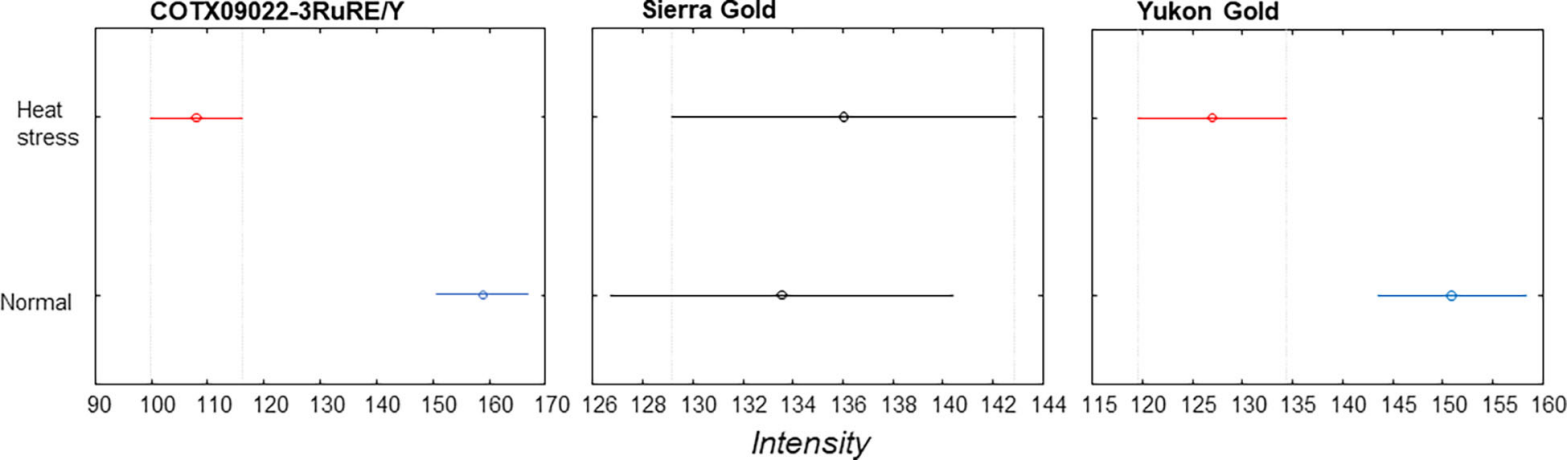
Mean (circles) and confidence interval (bars) at 1527 cm^-1^ (carotenoids) for three yellow flesh potato clones.

The averaged Raman spectra acquired from tubers of all potato clones grown under normal and heat-stressed conditions exhibit vibrational bands that could be assigned to starch, protein, carotenoids, cellulose and phenylpropanoids (**Figures 4**, **5**; **Table 4**). There was a significant decrease in the intensity of most vibrational bands that originated from carbohydrates (477 cm^-1^, 865 cm^-1^, 940 cm^-1^, 1085 cm^-1^, 1126 cm^-1^, 1261 cm^-1^ and 1340 cm^-1^) in Raman spectra acquired from tubers harvested from heat-stressed plants compared to the intensities of these bands in the Raman spectra collected from tubers of plants grown under normal conditions (**Figures 4**, **5**, **Supplementary Figure 2**). We also found changes in the intensities of bands that originated from proteins and phenylpropanoids in the spectra acquired from tubers of normal vs. heat-stressed plants. However, no significant changes were observed in the intensities of carotenoid bands (**Figure 6**). These results suggested that the concentration of carotenoids did not change in potato tubers due to the high-temperature stress. Thus, we can conclude that concentrations of starch, proteins and phenylpropanoids decreased in tubers produced by plants exposed to high-temperature stress. It should be noted that when the yellow flesh clones were compared individually, the carotenoid peaks were significantly more intense in the spectra collected from tubers from normal conditions versus those from heat stress conditions in COTX09022-3RuRE/Y and Yukon Gold, but no significant change in the intensity of carotenoids in the spectra acquired from another fresh yellow flesh clone Sierra Gold™, a heat-tolerant early variety from the Texas A&M Program (**Figure 7**).

**Table 4:** Chemical assignments based on the vibrational mode of each band-Raman shift (modified from Morey et al., 2020).

Band (cm^-1^)	Vibrational mode	Assignment (References)
441	Skeletal modes of pyranose ring	Carbohydrates (Kizil et al., 2002; Almeida et al., 2010)
479	C-C-O and C-C-C deformations; Related to glycosidic ring skeletal deformations δ(C-C-C) +τ(C-O) Scissoring of C-C-C and out-of-plane bending of C-O	Carbohydrates (Almeida et al., 2010)
523	δ(C-C-O) + τ(C-O) of carbohydrates	Carbohydrates (Almeida et al., 2010)
578	ν(C-O) +ν(C-C) +δ(C-O-H)	Cellulose, phenylpropanoids (Edwards et al., 1997)
615	δ(C-C-O) of carbohydrates	Carbohydrates (Almeida et al., 2010)
717	δ(C-C-O) related to glycosidic ring skeletal deformations	Carbohydrates (Almeida et al., 2010)
768	δ(C-C-O)	Carbohydrates (Almeida et al., 2010)
865	δ(C-C-H) +δ(C-O-C) glycosidic bond; anomeric region	Carbohydrates (Almeida et al., 2010)
940	Skeletal modes; δ(C-O-C) + δ(C-O-H) +v(C-O) α-1,4 glycosidic linkages	Carbohydrates (De Gussem et al., 2005)
1007	In-plane CH_3_ rocking + C-C	Carotenoids (Schulz et al., 2005)
1016	C-OH	Carbohydrates (Edwards et al., 1997)
1054	ν(C-O) +ν(C-C) +δ(C-O-H)	Carbohydrates (Almeida et al., 2010)
1084	ν(C-O) +ν(C-C) +δ(C-O-H)	Carbohydrates (Almeida et al., 2010)
1126	ν(C-O) +ν(C-C) +δ(C-O-H)	Carbohydrates (Almeida et al., 2010)
1153	ν(C-O-C), ν(C-C) in glycosidic linkage, asymmetric ring breathing	Carbohydrates (Wiercigroch et al., 2017)
1208	aromatic ring modes of phenylalanine and tyrosine; symmetric O-CH_3_ wag + C-O-H bending	Proteins (Zheng et al., 2004), Phenylpropanoids (Larsen and Barsberg, 2010)
1261	δ(C-C-H) +δ(O-C-H) +δ(C-O-H)	Carbohydrates (Cael et al., 1975; Almeida et al., 2010)
1340	ν(C-O); δ(C-O-H)	Carbohydrates (Almeida et al., 2010)
1383	δ(C-O-H) - coupling of the CCH and COH deformation modes	Carbohydrates (Almeida et al., 2010)
1398	δ(C-C-H)	Carbohydrates (Almeida et al., 2010)
1460	δ(CH)+δ(CH_2_) +δ(C-O-H) CH, CH_2_, and COH deformations.	Aliphatic (Almeida et al., 2010)
1530	-C=C-	Carotenoids (Adar, 2017)
1600	ν(C-C) aromatic ring + σ(CH)	Phenylpropanoids (Agarwal, 2006; Kang et al., 2016), proteins (Kang et al., 2016)
1633	C=C-C(ring)	Phenylpropanoids (Pompeu et al., 2018)
1660	amide I (C=O)	Proteins (Egging et al., 2018)

Next, PLS-DA was used to investigate the prediction accuracy of heat stress in potato clones grown in 2020 and 2021 (**Table 5**). We found that developed models could identify heat stress with accuracies of 63.8% in 2020 and 68.8% in 2021. At the same time, the absence of stress could be identified with accuracies of 73.4% in 2020 and 84.3% in 2021.

**Table 5:** Confusion matrix for all-potato cultivars evaluated under greenhouse conditions in 2020 and 2021 to determine if the environmental conditions in which potatoes were grown (normal *vs.* heat stress) can be predicted (true positive rate – TPR) based on Raman shifts.

	Predicted as normal	Predicted as heat stressed	Total number of scans	True Positive Rate (TPR)
2020				
** Normal**	270	96	368	73.4%
** Heat stressed**	98	169	265	63.8%
2021				
** Normal**	706	131	837	84.3%
** Heat stressed**	307	676	983	68.8%

## 4 Discussion

Changes observed in potato tubers (specific gravity, dry matter, reducing sugars, protein) harvested from plants grown under high-temperature stress compared to those harvested from potatoes grown under normal temperature were generally reflected in Raman scans. Raman spectroscopy captured changes in chemical composition for starch, phenylpropanoids, carotenoids, proteins, and other chemicals. The protein peaks had low intensity, likely because proteins were present in lower concentrations than starch.

### 4.1 Specific gravity

Having high specific gravity is very important in processing potatoes. In these experiments, Russet Burbank was used as a reference for processing French fries, and Atlantic as a reference for processing chippers. Processors are paid by the dry weight of the final product, and oil expenses are directly related to the amount of water replaced in tubers; thus, less water in tubers (higher specific gravity) is desired in processing potatoes. Sensory attributes for French fries and chippers are better in potatoes with high specific gravity (high dry matter, high starch). Heat stress has been reported to decrease the specific gravity of potatoes (Teixeira et al., 2015; Andrade et al., 2021; Fernandes Filho et al., 2021). The decrease in specific gravity is regarded as a loss to the processors. The decrease in specific gravity indicates that there is less dry matter accumulated in the potato tuber. A reduction in dry matter would result in reduced income. The effect of heat stress can be seen as a general decrease in specific gravity in all potato clones tested. The greatest decrease in specific gravity was observed in Russet Burbank, indicating that heat significantly reduced its processing quality and thus can be considered heat sensitive. Reveille Russet is a fresh market potato and has low specific gravity. The reduction of specific gravity in Reveille Russet due to heat stress was proportionally lower than that observed in Russet Burbank. The results of this experiment show that Atlantic when grown under normal (temperature) conditions, gives the highest specific gravity, but its specific gravity decreases when grown under high-temperature conditions indicating its sensitivity towards heat stress.

### 4.2 Tuber dry matter

Tuber dry matter of potatoes is an important quality characteristic for processing that can also be obtained from specific gravity (Nzaramba et al., 2013). Several reports have also indicated that tuber dry matter is drastically reduced when potatoes are grown above 20-25°C (Levy and Veilleux, 2007; Aien et al., 2017; Busse et al., 2018; Kim and Lee, 2019; Obiero et al., 2019). As a result of low dry matter, the quality of the chips and French fries decreases. The chips/fries absorb more oil when potatoes have lower dry matter, as indicated by low specific gravity. Similar to our results with specific gravity, the tuber dry matter also showed a decrease in potatoes grown under high temperatures compared to normal (temperature) conditions. Under similar heat stress temperatures of 35/25°C day/night, it was reported that high temperature caused a reduction in assimilate partitioning (radio labeling of ^14^C) to tubers in four clones tested. This reduction in partitioning was evident with a significant decrease in tuber dry matter percentage grown under high temperatures compared to Normal (25/12°C day/night) (Gawronska et al., 1992). The reduction of dry matter concentration under high temperatures has also been associated with the synthesis of high levels of endogenous gibberellins, which reduce the partitioning of assimilates to the tubers and impede the synthesis of starch and tuber-specific proteins (Lovell and Booth, 1967).

### 4.3 Reducing sugars

Temperature-induced increase in soluble sugars was observed in potatoes grown under moderately elevated temperatures in Chile (Ávila-Valdés et al., 2020). Soil temperatures of 23°C and 29°C during tuber bulking have been reported to increase the reducing sugars more than two folds, even in the Low-temperature sweetening-resistant clone Premier Russet (Zommick et al., 2014). Heat stress of just 14 days has been reported to increase the reducing sugar-glucose in potatoes (Snowden, Lamoka, Megachip and Nicolet), especially in the basal ends of tubers when grown under a high temperature of 35/29°C day/night.

### 4.4 Protein

Heat stress increased the protein concentration (DW) in the potatoes based on wet chemistry (**Figures 1**, **2**). When plants were grown at the high-temperature regime, a greater increase in protein concentration due to heat stress was found compared to the normal temperature regime. A decrease in individual tuber dry mass accompanied a large part of the increase found in protein concentration. Stone and Nicolas (1998) observed a similar case and reported that heat stress increased grain protein percentage even though the overall protein content per grain was reduced by heat.

Dry matter accumulation drastically decreased under heat stress. Since the average tuber dry mass decreased, it can be assumed that the negative effect of heat stress on starch biosynthesis led to a lower level or dilution of tuber protein. The tuber dry matter is mainly starch, and its greater reduction under heat stress would increase protein percent on a dry basis. On the other hand, the protein percentage of tubers, even if significantly higher at heated conditions than potatoes under normal temperature, is very small in comparison to potatoes’ starch. Thus, the observation that the protein content of potatoes under heat stress conditions is lower than that of normal conditions based on the Raman spectrum is contrary to our wet chemistry findings. Protein content in tubers based on wet chemistry was calculated from total nitrogen content. Tuber dry matter and other parameters decreased under heat stress conditions. Thus, it is likely that the absolute protein content per plot/clone did not change or even decrease despite the increase in percentage DW based on a tuber sample.

Clones varying in temperature tolerance showed differential responses with their root proteins. In a recent study, the sensitive clone showed a twofold increase in defense and detoxification-related proteins. In contrast, the tolerant clone showed more increase than decrease in proteins related to energy and carbohydrate metabolism (Boguszewska-Mańkowska et al., 2020). Stress-responsive proteins like HSP17, 6-CI, HSP101, and eEF1A are associated with microtubers under high-temperature stress. A higher level of eEF1A in the experiment was putatively marked as lowering the negative effects of heat in potato tuberization (Pantelić et al., 2018). It is plausible to think that the protein content of potatoes would increase at the expense of starch under stress. However, total soluble protein content (DW) was found not to be significantly different in potatoes grown under different temperature conditions (Ávila-Valdés et al., 2020). Also, the total protein content of strawberry plants decreased under heat stress conditions (Gulen and Eris, 2004).

### 4.5 Raman spectra

RS determined the effect of heat stress on different cultivars of potatoes. In most potatoes, the carbohydrate levels decreased when plants were grown under heat stress. Phenylpropanoids were significantly reduced in tubers produced under heat-stress conditions, but there was no significant reduction in carotenoids. The strongest Raman signal for starch around 477-479 cm^-1^ was used as a marker for the quantification of total cellular starch (Kizil et al., 2002; Ji et al., 2014; Morey et al., 2020). This main starch peak was also found in this experiment to be expressed at a significantly higher intensity in potatoes grown under normal conditions compared with the potatoes grown under higher temperatures. Raman spectra can reveal differences in the starch content of potatoes grown under different conditions, and Raman spectroscopy has the potential to estimate starch content in potatoes. In a previous study, Morey et al. (2020) verified that Raman spectroscopy can predict the starch content of potato samples based on the intensity of the 479 cm^-1^ band. Quantification of amylopectin and amylose, as well as proteins, have been achieved in rice (Pezzotti et al., 2021), protein, and oil in soybean (Singh et al., 2019) through the use of Raman spectroscopy. Tuber carotenoids do not seem to change in response to heat stress. Similar to our results with Raman spectra, Fogelman et al. (2019) reported that carotenoid levels did not change with the heat treatment (33-35°C) of tubers for one week before harvest. Also, no significant carotenoid changes were observed in heat stress-treated potato plants compared to normal by Shah et al. (2020). Thus, several studies showed evidence to conclude carotenoids are not being changed with heat stress.

On the other hand, phenylpropanoids showed a significant reduction under heat stress compared to normal (temperature) growing conditions. Secondary metabolites are affected by high-temperature stress. Their lower concentration in potatoes in the heated conditions means that either phenylpropanoids were used to protect other biochemical compounds like proteins and allow normal functioning of the tuber, or their biosynthetic pathway was disrupted by excess temperature. In both scenarios, phenylpropanoids seem to be lower under heat stress. A more detailed investigation would be required to pin down the types of phenylpropanoids significantly affected by growing conditions. Although several factors contribute to a location’s effect on a crop metabolite profile, the temperature seemed to be one of the factors to lower the total phenylpropanoids measured in Magic Molly potatoes across different sites in Alaska, Texas, and Florida (Payyavula et al., 2012). One of the most abundant phenylpropanoids, chlorogenic acid (CGA), decreased from locations in Alaska to Texas and Florida. Anthocyanin was found to decrease with increasing temperature. A higher level of total phenolics in potatoes was reported at a location with lower temperatures (Reddivari et al., 2007).

Also, RS predicted the tubers’ growing condition based on the model built with PLSDA. Since fewer good scans were obtained in 2020, a comparison of Raman spectra of individual cultivars and construction of confusion tables were generated for 2021 datasets only (**Table 6**). The percent accuracies in determining the presence of heat stress in several cultivars (Reveille Russet and Vanguard Russet) was low, indicating that these cultivars were not predicted as heat-sensitive cultivars (thus, declaring them as heat tolerant).

**Table 6:** Confusion matrix for individual potato cultivars evaluated under greenhouse conditions in 2021 to determine if the environmental conditions in which potatoes were grown (normal vs. heat stress) can be predicted (true positive rate - TPR) based on Raman shifts.

Clone (Variety)	Stress	TPR (%)
Atlantic	Normal	80.3
	Heat stress	75.6
COTX09022-3RuRE/Y	Normal	86.5
	Heat stress	77.1
Reveille Russet	Normal	87.1
	Heat stress	68.5
Russet Burbank	Normal	83.3
	Heat stress	81.3
Russet Norkotah	Normal	86.9
	Heat stress	73.5
Russet Norkotah278	Normal	87.9
	Heat stress	90.8
Russet Norkotah296	Normal	91.7
	Heat stress	86.2
Sierra Gold™	Normal	78.7
	Heat stress	78.1
Vanguard Russet	Normal	82.0
	Heat stress	68.1
Yukon Gold	Normal	79.1
	Heat stress	78.6

The difference in TPR for different cultivars could be inspected to see that some potato clones were more tolerant to heat stress than others. Similar TPR % for both growing conditions when considering a clone indicates that the model could distinguish between the treatments; however, a large difference in the TPR% between the conditions indicates that the spectrum of one condition overlapped with the other. The lower TPR % for clones like Reveille Russet and Vanguard Russet indicated that their spectra of potatoes grown under heated conditions overlapped with that of potatoes under normal conditions, indicating their chemical signatures are similar. This spectrum similarity under different conditions means that the potatoes of these cultivars behaved similarly to the conditions. Thus, we could assign them to be more heat tolerant than others of heat stress. Similarly, heat-susceptible clones like Atlantic and Russet Burbank could be differentiated with greater confidence for their growth condition. They exhibited different signature spectrums under different conditions when scanned with Raman.

## 5 Conclusion

In general, Raman spectroscopy could differentiate the chemical composition of tubers based on the conditions in which potato plants were grown (normal vs. heat stress). The intensities of vibrational bands corresponding to carbohydrates and phenypropanoids were the most significantly reduced under heat-stress. However, heat-tolerant clones could be identified based on having similar (not significantly different) intensities of vibrational bands independently of the growing conditions (normal *vs*. heat stress). Raman spectra-based prediction TPR (True Positive Rate) values > 70-80% could aid potato breeding programs by identifying heat-sensitive clones. In contrast, if the TPR values are lower, it would indicate that the clones are heat tolerant. We foresee the application of Raman spectroscopy to study tolerance to abiotic (heat, drought, cold, salinity) and biotic (bacterial, fungal, nematodes, insects) stresses based on the chemical changes the stresses induce (in potato tubers, plants, or seeds – in other crops-). Additional areas of expansion could include the study of chemical changes in produce postharvest in response to different storage conditions, presence of storage diseases/pests, and in response to different storage periods.

## Data availability statement

The original contributions presented in the study are included in the article/**Supplementary Material**. Further inquiries can be directed to the corresponding author/s.

## Author contributions

MIV, DS, SG, and DK conceived and designed the experiments, supervised experiments, and guided the data analysis. DS, SG, NR, and RM performed experiments and analyzed data. All authors contributed to the article and approved the submitted version.

## References

[B1] Adar F. (2017). Carotenoids-their resonance raman spectra and how they can be helpful in characterizing a number of biological systems. Spectroscopy 32, 12–20.

[B2] Agarwal U. P. (2006). Raman imaging to investigate ultrastructure and composition of plant cell walls: Distribution of lignin and cellulose in black spruce wood (Picea mariana). Planta 224, 1141–1153. doi: 10.1007/s00425-006-0295-z 16761135

[B3] Aien A. Chaturvedi A. K. Bahuguna R. N. Pal M. (2017). Phenological sensitivity to high temperature stress determines dry matter partitioning and yield in potato. Indian J. Plant Physiol. 22, 63–69. doi: 10.1007/s40502-016-0270-z

[B4] Almeida M. R. Alves R. S. Nascimbem L. B. L. R. Stephani R. Poppi R. J. de Oliveira L. F. C. (2010). Determination of amylose content in starch using raman spectroscopy and multivariate calibration analysis. Anal. Bioanal. Chem. 397, 2693–2701. doi: 10.1007/s00216-010-3566-2 20213166

[B5] Andrade M. H. M. L. Patiño-Torres A. J. Cavallin I. C. Guedes M. L. Carvalho R. P. Gonçalves F. M. A.. (2021). Stability of potato clones resistant to potato virus y under subtropical conditions. Crop Breed. Appl. Biotechnol. 21, 1–9. doi: 10.1590/1984-70332021v21n1a8

[B6] Asmamaw Y. Tekalign T. Workneh T. S. (2010). Specific gravity, dry matter concentration, pH, and crisp-making potential of Ethiopian potato (Solanum tuberosum l.) cultivars as influenced by growing environment and length of storage under ambient conditions. Potato. Res. 53, 95–109. doi: 10.1007/s11540-010-9154-1

[B7] Ávila-Valdés A. Quinet M. Lutts S. Martínez J. P. Lizana X. C. (2020). Tuber yield and quality responses of potato to moderate temperature increase during tuber bulking under two water availability scenarios. F. Crop Res. 251, 107786. doi: 10.1016/j.fcr.2020.107786

[B8] Baranska M. Schütze W. Schulz H. (2006). Determination of lycopene and β-carotene content in tomato fruits and related products: Comparison of FT-raman, ATR-IR, and NIR spectroscopy. Anal. Chem. 78, 8456–8461. doi: 10.1021/ac061220j 17165839

[B9] Bashir I. Nardino M. Castro C. M. Heiden G. (2022). Genotypic response and selection of potato germplasm under heat stress. Potato. Res. 66, 85–104. doi: 10.1007/s11540-022-09573-w

[B10] Beals K. A. (2019). Potatoes, nutrition and health. Am. J. Potato. Res. 96, 102–110. doi: 10.1007/s12230-018-09705-4

[B11] Berardo N. Brenna O. V. Amato A. Valoti P. Pisacane V. Motto M. (2004). Carotenoids concentration among maize genotypes measured by near infrared reflectance spectroscopy (NIRS). Innov. Food Sci. Emerg. Technol. 5, 393–398. doi: 10.1016/j.ifset.2004.03.001

[B12] Boguszewska-Mańkowska D. Gietler M. Nykiel M. (2020). Comparative proteomic analysis of drought and high temperature response in roots of two potato cultivars. Plant Growth Regul. 92, 345–363. doi: 10.1007/s10725-020-00643-y

[B13] Bonnel E. (2008). Potato breeding: a challenge, as ever! Potato. Res. 51, 327–332. doi: 10.1007/s11540-008-9116-z

[B14] Bushnell J. (1925). The relation of temperature to growth and respiration in the potato plant. Am. Potato. J. 4, 119–119. doi: 10.1007/BF02910567

[B15] Busse J. S. Wiberley-Bradford A. E. Bethke P. C. (2018). Transient heat stress during tuber development alters post-harvest carbohydrate composition and decreases processing quality of chipping potatoes. J. Sci. Food Agric. 99, 2579–2588. doi: 10.1002/jsfa.9473 30411360

[B16] Cael J. J. Koenig J. L. Blackwell J. (1975). Infrared and raman spectroscopy of carbohydrates. part VI: Normal coordinate analysis of V-amylose. Biopolymers 14, 1885–1903. doi: 10.1002/bip.1975.360140909

[B17] CIP (2020) International potato center - potato facts and figures (Int. Potato Cent.). Available at: https://cipotato.org/potato/potato-facts-and-figures/ (Accessed May 20, 2022).

[B18] Damodaran S. Parkin K. L. Fennema O. (2017). Fennema’s food chemistry, fifth edition. 5th ed. Eds. Damodaran S. Parkin K. L. (Boca Raton, FL,USA: CRC Press). doi: 10.1201/9781315372914

[B19] De Gussem K. Vandenabeele P. Verbeken A. Moens L. (2005). Raman spectroscopic study of lactarius spores (Russulales, fungi). Spectrochim. Acta Part A Mol. Biomol. Spectrosc. 61, 2896–2908. doi: 10.1016/J.SAA.2004.10.038 16165029

[B20] Demirel U. Çalişkan S. Yavuz C. Tindaş İ. Polgar Z. Vaszily Z.. (2017). Assessment of morphophysiological traits for selection of heat-tolerant potato genotypes. Turkish. J. Agric. For. 41, 218–232. doi: 10.3906/tar-1701-95

[B21] Edwards H. G. M. Farwell D. W. Webster D. (1997). FT raman microscopy of untreated natural plant fibres. Spectrochim. Acta Part A Mol. Biomol. Spectrosc. 53, 2383–2392. doi: 10.1016/S1386-1425(97)00178-9 9477578

[B22] Egging V. Nguyen J. Kurouski D. (2018). Detection and identification of fungal infections in intact wheat and sorghum grain using a hand-held raman spectrometer. Anal. Chem. 90, 8616–8621. doi: 10.1021/acs.analchem.8b01863 29898358

[B23] Eldredge E. P. Holmes Z. A. Mosley A. R. Shock C. C. Stieber T. D. (1996). Effects of transitory water stress on potato tuber stem-end reducing sugar and fry color. Am. Potato. J. 73, 517–530. doi: 10.1007/BF02851697

[B24] FAO (2019) FAO global statistical Yearbook, FAO regional statistical yearbooks (FAOSTAT). Available at: http://www.fao.org/faostat/en/#data/QC (Accessed May 4, 2021).

[B25] FAO/WHO (1998). “Dietary fibre and resistant starch analysis,” in Carbohydrates in human nutrition (Rome: World Health Organization), 11–14. Available at: http://www.fao.org/docrep/w8079e/w8079e0a.htm.

[B26] FAOSTAT (2022) (Food and Agriculture Organization of the United Nations). Available at: https://www.fao.org/faostat/en/#home (Accessed October 12, 2022).

[B27] Farber C. Sanchez L. Pant S. Scheuring D. Vales I. Mandadi K.. (2021). Potential of spatially offset raman spectroscopy for detection of zebra chip and potato virus y diseases of potatoes (Solanum tuberosum). ACS Agric. Sci. Technol. 1, 211–221. doi: 10.1021/acsagscitech.1c00024

[B28] Fernandes Filho C. C. Andrade M. H. M. L. Souza Marçal T. Fernandes M. O. Bastos A. J. R. Guedes M. L.. (2021). Selection of potato clones for heat tolerance and resistance to potato viruses X and y for processing purposes. Crop Sci. 61, 552–565. doi: 10.1002/csc2.20361

[B29] Fogelman E. Oren-Shamir M. Hirschberg J. Mandolino G. Parisi B. Ovadia R.. (2019). Nutritional value of potato (Solanum tuberosum) in hot climates: Anthocyanins, carotenoids, and steroidal glycoalkaloids. Planta 249, 1143–1155. doi: 10.1007/s00425-018-03078-y 30603793

[B30] Fumia N. Pironon S. Rubinoff D. Khoury C. K. Gore M. A. Kantar M. B. (2022). Wild relatives of potato may bolster its adaptation to new niches under future climate scenarios. Food Energy Secur 11, 1–15. doi: 10.1002/fes3.360

[B31] Gautam S. Solis-Gracia N. Teale M. K. Mandadi K. da Silva J. A. Vales M. I. (2021). Development of an in vitro microtuberization and temporary immersion bioreactor system to evaluate heat stress tolerance in potatoes (Solanum tuberosum l.). Front. Plant Sci. 12. doi: 10.3389/fpls.2021.700328 PMC8385365 34456944

[B32] Gawronska H. Thornton M. K. Dwelle R. B. (1992). Influence of heat stress on dry matter production and photo-assimilate partitioning by four potato clones. Am. Potato. J. 69, 653–665. doi: 10.1007/BF02852678

[B33] Gonçalves C. Rodriguez-Jasso R. M. Gomes N. Teixeira J. A. Belo I. (2010). Adaptation of dinitrosalicylic acid method to microtiter plates. Anal. Methods 2, 2046. doi: 10.1039/c0ay00525h

[B34] Gould W. A. Plimpton S. (1985). Quality evaluation of potato cultivars. North Cent. Reg. Res. Publ. 305, 1–25.

[B35] Gulen H. Eris A. (2004). Effect of heat stress on peroxidase activity and total protein content in strawberry plants. Plant Sci. 166, 739–744. doi: 10.1016/j.plantsci.2003.11.014

[B36] Haverkort A. J. Linnemann A. R. Struik P. C. Wiskerke J. S. C. (2022). On processing potato. 4. survey of the nutritional and sensory value of products and dishes (Netherlands: Springer). doi: 10.1007/s11540-022-09568-7

[B37] Haverkort A. J. Verhagen A. (2008). Climate change and its repercussions for the potato supply chain. Potato. Res. 51, 223–237. doi: 10.1007/s11540-008-9107-0

[B38] Hijmans R. J. (2003). The effect of climate change on global potato production. Am. J. Potato. Res. 80, 271–279. doi: 10.1007/BF02855363

[B39] IPCC (2021). Climate Change 2021: The Physical Science Basis. In Eds. Masson-Delmotte V. Zhai P. Pirani A. Connors S. L. Péan C. Berger S. Contribution of Working Group I to the Sixth Assessment Report of the Intergovernmental Panel on Climate Change. Cambridge, United Kingdom and New York, NY, USA: Cambridge University Press. doi: 10.1017/9781009157896

[B40] Islam M. M. Naznin S. Naznin A. Uddin M. N. Amin M. N. Rahman M. M.. (2022). Dry matter, starch content, reducing sugar, color and crispiness are key parameters of potatoes required for chip processing. Horticulturae 8, 1–12. doi: 10.3390/horticulturae8050362

[B41] Ji Y. He Y. Cui Y. Wang T. Wang Y. Li Y.. (2014). Raman spectroscopy provides a rapid, non-invasive method for quantitation of starch in live, unicellular microalgae. Biotechnol. J. 9, 1512–1518. doi: 10.1002/biot.201400165 24906189

[B42] Kang L. Wang K. Li X. Zou B. (2016). High pressure structural investigation of benzoic acid: Raman spectroscopy and X-ray diffraction. J. Phys. Chem. C 120, 14758–14766. doi: 10.1021/acs.jpcc.6b05001

[B43] Kim Y. U. Lee B.-W. (2019). Differential mechanisms of potato yield loss induced by high day and night temperatures during tuber initiation and bulking: Photosynthesis and tuber growth. Front. Plant Sci. 10. doi: 10.3389/fpls.2019.00300 PMC6426786 30923532

[B44] Kim Y. Singh M. Kays S. (2007). Near-infrared spectroscopic analysis of macronutrients and energy in homogenized meals. Food Chem. 105, 1248–1255. doi: 10.1016/j.foodchem.2007.03.011

[B45] Kizil R. Irudayaraj J. Seetharaman K. (2002). Characterization of irradiated starches by using FT-raman and FTIR spectroscopy. J. Agric. Food Chem. 50, 3912–3918. doi: 10.1021/jf011652p 12083858

[B46] Kurouski D. Van Duyne R. P. Lednev I. K. (2015). Exploring the structure and formation mechanism of amyloid fibrils by raman spectroscopy: A review. Analyst 140, 4967–4980. doi: 10.1039/c5an00342c 26042229

[B47] Larsen K. L. Barsberg S. (2010). Theoretical and raman spectroscopic studies of phenolic lignin model monomers. J. Phys. Chem. B 114, 8009–8021. doi: 10.1021/jp1028239 20499919

[B48] Levy D. Veilleux R. E. (2007). Adaptation of potato to high temperatures and salinity-a review. Am. J. Potato. Res. 84, 487–506. doi: 10.1007/BF02987885

[B49] Lovell P. H. Booth A. (1967). Effects of gibberellic acid on growth, tuber formation and carbohydrate distribution in solanum tuberosum. New Phytol. 66, 525–537. doi: 10.1111/j.1469-8137.1967.tb05424.x

[B50] Lulai E. C. Orr P. H. (1979). Influence of potato specific gravity on yield and oil content of chips. Am. Potato. J. 56, 379–390. doi: 10.1007/BF02855348

[B51] Matousek P. Morris M. D. Everall N. Clark I. P. Towrie M. Draper E.. (2005). Numerical simulations of subsurface probing in diffusely scattering media using dpatially offset raman spectroscopy. Appl. Spectrosc. 59, 1485–1492. doi: 10.1366/000370205775142548 16390587

[B52] McGeehan S. L. Naylor D. V. (1988). Automated instrumental analysis of carbon and nitrogen in plant and soil samples. Commun. Soil Sci. Plant Anal. 19, 493–505. doi: 10.1080/00103628809367953

[B53] McGill C. R. Kurilich A. C. Davignon J. (2013). The role of potatoes and potato components in cardiometabolic health: A review. Ann. Med. 45, 467–473. doi: 10.3109/07853890.2013.813633 23855880

[B54] Mihaljev Ž.A. Jakšić S. M. Prica N. B. Ćupić Ž.N. Živkov-Baloš M. M. (2015). Comparison of the kjeldahl method, Dumas method and NIR method for total nitrogen determination in meat and meat products. J. Agroaliment. Process. Technol. 21, 365–370.

[B55] Morey R. Ermolenkov A. Payne W. Z. Scheuring D. C. Koym J. W. Vales M. I.. (2020). Non-invasive identification of potato varieties and prediction of the origin of tuber cultivation using spatially offset raman spectroscopy. Anal. Bioanal. Chem. 412, 4585–4594. doi: 10.1007/s00216-020-02706-5 32451641

[B56] Nzaramba M. N. Scheuring D. C. Koym J. W. Miller J. C. (2013). Relationships among antioxidant activity, total phenolic content and specific gravity in several potato (Solanum tuberosum l.) cultivars grown in different environments. Am. J. Potato. Res. 90, 541–550. doi: 10.1007/s12230-013-9326-z

[B57] Obiero C. O. Milroy S. P. Bell R. W. (2019). Importance of whole plant dry matter dynamics for potato (Solanum tuberosum l.) tuber yield response to an episode of high temperature. Environ. Exp. Bot. 162, 560–571. doi: 10.1016/j.envexpbot.2019.04.001

[B58] Osborne B. G. Fearn T. Hindle P. H. (1993). Practical NIR spectroscopy with applications in food and beverage analysis. Eds. Osborne B. G. Fearn T. Hindle P. H. (Harlow,UK: Addison-Wesley Longman Ltd).

[B59] Pantelić D. Dragićević I. Rudić J. Fu J. Momčilović I. (2018). Effects of high temperature on *in vitro* tuberization and accumulation of stress-responsive proteins in potato. Hortic. Environ. Biotechnol. 59, 315–324. doi: 10.1007/s13580-018-0043-x

[B60] Payyavula R. S. Navarre D. A. Kuhl J. C. Pantoja A. Pillai S. S. (2012). Differential effects of environment on potato phenylpropanoid and carotenoid expression. BMC Plant Biol. 12, 1–17. doi: 10.1186/1471-2229-12-39 22429339 PMC3342224

[B61] Pezzotti G. Zhu W. Chikaguchi H. Marin E. Boschetto F. Masumura T.. (2021). Raman molecular fingerprints of rice nutritional quality and the concept of raman barcode. Front. Nutr. 8. doi: 10.3389/fnut.2021.663569 PMC8260989 34249986

[B62] Pompeu D. R. Larondelle Y. Rogez H. Abbas O. Pierna J. A. F. Baeten V. (2018). Characterization and discrimination of phenolic compounds using Fourier transform raman spectroscopy and chemometric tools. BASE 22, 13–28. doi: 10.25518/1780-4507.16270

[B63] Reddivari L. Hale A. L. Miller J. C. (2007). Genotype, location, and year influence antioxidant activity, carotenoid content, phenolic content, and composition in specialty potatoes. J. Agric. Food Chem. 55, 8073–8079. doi: 10.1021/jf071543w 17760413

[B64] Sanchez L. Ermolenkov A. Biswas S. Septiningsih E. M. Kurouski D. (2020). Raman spectroscopy enables non-invasive and confirmatory diagnostics of salinity stresses, nitrogen, phosphorus, and potassium deficiencies in rice. Front. Plant Sci. 11. doi: 10.3389/fpls.2020.573321 PMC7642205 33193509

[B65] SAS Institute Inc (2021). JMP®16 documentation library (Cary, NC: SAS Institute Inc).

[B66] Schulz H. Baranska M. Baranski R. (2005). Potential of NIR-FT-Raman spectroscopy in natural carotenoid analysis. Biopolymers 77, 212–221. doi: 10.1002/BIP.20215 15674976

[B67] Shah Z. Shah S. H. Ali G. S. Munir I. Khan R. S. Iqbal A.. (2020). Introduction of arabidopsis’s heat shock factor HsfA1d mitigates adverse effects of heat stress on potato (Solanum tuberosum l.) plant. Cell Stress Chaperones 25, 57–63. doi: 10.1007/s12192-019-01043-6 31898287 PMC6985360

[B68] Singh R. Wrobel T. P. Mukherjee P. Gryka M. Kole M. Harrison S.. (2019). Bulk protein and oil prediction in soybeans using transmission raman spectroscopy: A comparison of approaches to optimize accuracy. Appl. Spectrosc. 73, 687–697. doi: 10.1177/0003702818815642 30409030

[B69] Stark J. C. (2020). Potato production systems. In: Eds. Stark J. C. Thornton M. Nolte P. Cham: Springer International Publishing. doi: 10.1007/978-3-030-39157-7

[B70] Stone P. J. Nicolas M. E. (1998). The effect of duration of heat stress during grain filling on two wheat varieties differing in heat tolerance: Grain growth and fractional protein accumulation. Funct. Plant Biol. 25, 13. doi: 10.1071/PP96114

[B71] Stubbs T. L. Kennedy A. C. Fortuna A. M. (2010). Using NIRS to predict fiber and nutrient content of dryland cereal cultivars. J. Agric. Food Chem. 58, 398–403. doi: 10.1021/jf9025844 19961223

[B72] Teixeira A. L. Pinto C. A. B. P. Lepre A. L. Peixouto L. S. Ribeiro G. H. M. R. (2015). Evaluation of potato clones for heat tolerance in the southern region of minas gerais, Brazil. Rev. Bras. Ciências. Agrárias. -. Braz. J. Agric. Sci. 10, 171–177. doi: 10.5039/agraria.v10i2a3268

[B73] Tito R. Vasconcelos H. L. Feeley K. J. (2018). Global climate change increases risk of crop yield losses and food insecurity in the tropical Andes. Glob. Change Biol. 24, e592–e602. doi: 10.1111/gcb.13959 29055170

[B74] USDA (2018) FoodData central. Available at: https://fdc.nal.usda.gov/fdc-app.html#/food-details/170026/nutrients (Accessed May 20, 2022).

[B75] Vales I. Miller C. Koym J. Scheuring D. (2019) Texas Potato breeding report 2018. Available at: http://potato.tamu.edu/reports.html.

[B76] Wiercigroch E. Szafraniec E. Czamara K. Pacia M. Z. Majzner K. Kochan K.. (2017). Raman and infrared spectroscopy of carbohydrates: A review. Spectrochim. Acta Part A Mol. Biomol. Spectrosc. 185, 317–335. doi: 10.1016/j.saa.2017.05.045 28599236

[B77] Zheng R. Zheng X. Dong J. Carey P. R. (2004). Proteins can convert to β-sheet in single crystals. Protein Sci. 13, 1288–1294. doi: 10.1110/ps.03550404 15096634 PMC2286773

[B78] Zhu T. Jackson D. S. Wehling R. L. Geera B. (2008). Comparison of amylose determination methods and the development of a dual wavelength iodine binding technique. Cereal Chem. J. 85, 51–58. doi: 10.1094/CCHEM-85-1-0051

[B79] Zommick D. H. Knowles L. O. Pavek M. J. Knowles N. R. (2014). In-season heat stress compromises postharvest quality and low-temperature sweetening resistance in potato (Solanum tuberosum l.). Planta 239, 1243–1263. doi: 10.1007/s00425-014-2048-8 24615233

