# Supplementary material for: Raman spectroscopy detects chemical differences between potato tubers produced under normal and heat stress growing conditions
Source: Front Plant Sci. 2023 Feb 23;14:1105603. doi: 10.3389/fpls.2023.1105603 (PMC9995913; doi:10.3389/fpls.2023.1105603)
Supplement: Supplementary file 1 [file DataSheet_1.docx]

**Raman Spectroscopy Detects Chemical Differences between Potato Tubers Produced under Normal and Heat stress Growing Conditions**

**Sanjeev Gautam^1^, Rohini Morey^2^, Nina Rau^1^, Douglas C. Scheuring^1^, Dmitry Kurouski^2,^*, M. Isabel Vales^1,^***

^1^ Department of Horticultural Sciences, Texas A&M University, College Station, TX, United States

^2^ Department of Biochemistry and Biophysics, Texas A&M University, College Station, TX, United States

*** Correspondence:**

Corresponding Authors

[dkurouski@tamu.edu](mailto:dkurouski@tamu.edu)

[isabel.vales@tamu.edu](mailto:isabel.vales@tamu.edu)

Supporting Information

Supplementary materials

**SUPPLEMENTARY TABLE 1|** Number of stressful days in greenhouses for years 2020 and 2021.

|  | **2020** | | **2021** | |
| --- | --- | --- | --- | --- |
| **Temperature conditions defining stress** | **Normal** | **Heat stress** | **Normal** | **Heat stress** |
| Stressful days with day temp.>35 and night temp.>25 | 0 | 2 | 0 | 25 |
| Stressful days with day temp.>30 and night temp.>25 | 1 | 2 | 0 | 26 |
| Stressful days with day temp.>35 and night temp.>20 | 1 | 19 | 9 | 55 |
| Stressful days with day temp.>30 and night temp.>20 | 11 | 24 | 34 | 66 |

**SUPPLEMENTARY TABLE 2|** Least squares (LS) Means for differents traits evaluated for effect of temperature conditions on ten potato clones (the trait means and significant differences between means were included in Figures 1 and 2).

|  |  | **Year 2020** | | | |  | **Year 2021** | | | |
| --- | --- | --- | --- | --- | --- | --- | --- | --- | --- | --- |
| **Clone** | **Condition** | **Specific gravity** | **Tuber dry matter** | **Reducing sugars** | **Protein %** |  | **Specific gravity** | **Tuber dry matter** | **Reducing sugars** | **Protein %** |
| Atlantic | Heat stress | 1.075 | 20.8 | 108.0 | 11.1 |  | 1.063 | 19.5 | 218.4 | 10.7 |
| COTX09022-3RuRE/Y | Heat stress | 1.048 | 18.1 | 289.2 | 11.4 |  | 1.048 | 16.6 | 720.9 | 10.5 |
| Reveille Russet | Heat stress | 1.048 | 18.8 | 478.3 | 12.4 |  | 1.039 | 17.0 | 1086.0 | 12.0 |
| Russet Burbank | Heat stress | 1.051 | 17.5 | 374.9 | 10.0 |  | 1.041 | 16.5 | 1126.8 | 9.9 |
| Russet Norkotah | Heat stress | 1.055 | 17.3 | 493.7 | 12.0 |  | 1.047 | 18.4 | 620.1 | 11.8 |
| Russet Norkotah278 | Heat stress | 1.055 | 17.0 | 327.7 | 11.0 |  | 1.047 | 18.1 | 652.0 | 11.8 |
| Russet Norkotah296 | Heat stress | 1.053 | 16.3 | 334.7 | 12.4 |  | 1.050 | 19.9 | 587.7 | 10.8 |
| Sierra Gold | Heat stress | 1.059 | 18.2 | 255.1 | 10.6 |  | 1.058 | 18.9 | 223.5 | 10.3 |
| Vanguard Russet | Heat stress | 1.051 | 17.2 | 401.2 | 11.3 |  | 1.046 | 15.7 | 627.3 | 11.5 |
| Yukon Gold | Heat stress | 1.069 | 19.2 | 364.7 | 10.7 |  | 1.065 | 19.7 | 448.3 | 10.5 |
| Atlantic | Normal | 1.086 | 23.2 | 97.9 | 9.3 |  | 1.082 | 23.3 | 131.8 | 8.7 |
| COTX09022-3RuRE/Y | Normal | 1.069 | 18.4 | 270.6 | 9.9 |  | 1.067 | 21.1 | 227.9 | 8.8 |
| Reveille Russet | Normal | 1.059 | 17.1 | 439.8 | 10.2 |  | 1.053 | 18.3 | 321.8 | 10.6 |
| Russet Burbank | Normal | 1.068 | 20.4 | 177.6 | 9.1 |  | 1.071 | 21.7 | 388.1 | 7.3 |
| Russet Norkotah | Normal | 1.065 | 20.5 | 182.6 | 11.3 |  | 1.074 | 21.3 | 257.1 | 10.3 |
| Russet Norkotah278 | Normal | 1.064 | 20.0 | 202.8 | 11.0 |  | 1.063 | 21.3 | 252.4 | 9.3 |
| Russet Norkotah296 | Normal | 1.065 | 19.5 | 240.7 | 11.1 |  | 1.066 | 21.3 | 296.6 | 9.1 |
| Sierra Gold | Normal | 1.072 | 21.3 | 130.7 | 9.4 |  | 1.073 | 21.9 | 217.7 | 8.2 |
| Vanguard Russet | Normal | 1.063 | 16.8 | 334.4 | 10.8 |  | 1.061 | 18.9 | 224.1 | 10.3 |
| Yukon Gold | Normal | 1.084 | 20.5 | 143.7 | 10.1 |  | 1.079 | 22.2 | 186.5 | 9.2 |

**SUPPLEMENTARY FIGURE 1|** Temperature conditions in greenhouses during 2020 and 2021.

**SUPPLEMENTARY FIGURE 2|** Raman spectra of tubers from ten potato cultivars grown under normal vs. heat stress greenhouse conditions (2020 and 2021). 2020 spectra were ‘offset’ (with the skin on), whereas 2021 spectra were ‘surface’ (a small section of the tuber skin was removed).
